# Glass-Based 4-in-1 High-Voltage Micro-LED Package for High-Brightness Mini-LED Backlight Applications

**DOI:** 10.3390/nano15231818

**Published:** 2025-12-01

**Authors:** Chien-Chi Huang, Tzu-Yi Lee, Chia-Hung Tsai, Fang-Chung Chen, Li-Yin Chen, Hao-Chung Kuo

**Affiliations:** 1 Department of Photonics, College of Electrical and Computer Engineering, National Yang Ming Chiao Tung University, Hsinchu 30010, Taiwan; cchuang.ee13@nycu.edu.tw (C.-C.H.); sppsai.ee12@nycu.edu.tw (C.-H.T.); fcchendop@nycu.edu.tw (F.-C.C.); lychen@nycu.edu.tw (L.-Y.C.); 2 Semiconductor Research Center, Hon Hai Research Institute, Taipei 11492, Taiwan; 3 Smartkem Ltd., Manchester M9 8GQ, UK; 4 Center for Emergent Functional Matter Science, National Yang Ming Chiao Tung University, Hsinchu 30010, Taiwan

**Keywords:** MicroLED-in-Package (MiP4), redistribution layer (RDL), glass substrate packaging

## Abstract

A novel four-in-one (4-in-series) MicroLED-in-Package (MiP4) architecture is demonstrated for the first time, integrating four sub-85 µm blue micro-LED (µ-LED) dies on a transparent glass substrate through a redistribution-layer (RDL) interconnection process. The MiP4 device operates natively at 16 V, eliminating the need for step-down converters and simplifying high-voltage backlight driving circuits. The transparent glass carrier enables efficient light extraction, excellent thermal dissipation, and uniform emission. Electrical and optical characterization of dual- (B2), triple- (B3), and quad-chip (B4) devices shows ideal voltage scalability (8 V, 12 V, 16 V) and stable emission at 450 ± 2 nm with minimal FWHM broadening (22–29 nm). Compared with a commercial LED, the MiP4 delivers 1.8× higher optical power (~41.8 mW) despite its active area being only ~1/70 that of the reference device (20,000 µm^2^ vs. 1,350,000 µm^2^), yielding a dramatically enhanced luminous flux density of 64 lm/mm^2^ at 50 mA. Furthermore, pulse-driven measurements under 2%, 5%, and 10% duty cycles verify excellent thermal stability and minimal spectral shift (<1 nm), confirming the device’s robustness and energy efficiency. This first-of-its-kind 4-in-1 high-voltage glass-based µ-LED package provides a scalable and manufacturable route toward next-generation ultra-thin, high-brightness Mini-LED backlight and optical communication systems.

## 1. Introduction

Mini light-emitting diode (Mini-LED) backlight technology has rapidly emerged as a pivotal innovation in modern display systems, delivering high contrast ratios, localized dimming capability, and enhanced color reproduction compared to conventional liquid-crystal-based solutions [1,2,3,4,5,6]. These advantages have made Mini-LED backlights indispensable in premium televisions, notebooks, and tablets. However, as display manufacturers strive for thinner form factors, higher resolution, and lower cost, the physical and electrical limitations of current Mini-LED architectures are becoming increasingly pronounced [7,8,9,10]. The dominant Mini-LED, featuring chip dimensions of 0.2 mm × 0.5 mm, exemplifies the challenges facing the next generation of high-density backlight units (BLUs). The relatively large chip footprint limits pixel density and prevents further reduction in optical thickness, both of which are critical for achieving finer dimming zones and slimmer backlight modules [11,12,13]. Moreover, to ensure uniform brightness over a wide emission angle, multiple optical films are typically incorporated in the backlight stack [14,15]. While these films enhance light distribution, they unavoidably lower luminous efficiency and increase manufacturing complexity and cost.

From a system-level standpoint, each dimming zone in a typical Mini-LED backlight integrates four to nine discrete chips to meet the required brightness [7,8,16,17,18]. This multi-chip configuration significantly increases the time required for pick-and-place operations during surface-mount assembly, thereby reducing throughput and increasing production costs. Compounding these issues is the electrical incompatibility between the 16 V driving voltage of conventional backlight driver circuits and the 3 V forward voltage of standard LED chips [6,19,20]. To bridge this mismatch, voltage conversion circuits are required, which introduce additional power loss, thermal management challenges, and layout complexity in the driver board design [21,22,23]. To overcome these persistent obstacles, this study proposes a novel packaging architecture designated as MiP4 (MicroLED-in-Package, four-in-series). The MiP4 configuration connects four sub-85 µm micro-LED (µ-LED) dies in series on a single glass-based carrier through advanced redistribution-layer (RDL) interconnect technology. This approach yields a compact 0.6 mm × 0.6 mm unit that operates natively at 16 V, effectively eliminating the need for step-down converters. The higher operating voltage not only improves power efficiency but also simplifies the driving circuitry. Additionally, the transparent glass substrate facilitates superior light extraction and broad emission profiles, enabling the MiP4 to deliver uniform illumination without relying on secondary optical diffusers. The system-level benefits of the MiP4 architecture extend beyond electrical simplification. By consolidating four μ-LEDs into a single package, the number of chips required per dimming zone is significantly reduced, streamlining the surface-mount-technology (SMT) process and enhancing assembly yield. The glass carrier and RDL layout also allow precise chip placement and efficient thermal dissipation, supporting large-scale array integration with excellent uniformity. These attributes make the MiP4 platform inherently scalable and compatible with high-throughput manufacturing of fine-pitch Mini-LED backlight modules.

**Figure 1:**
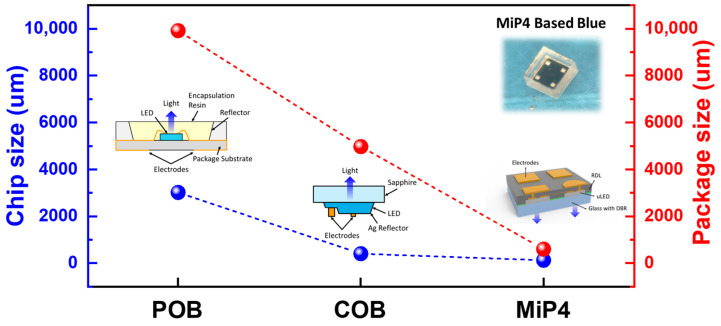
Comparison of chip (blue) and package (red) dimensions for three backlight packaging architectures: POB, COB, and the proposed MiP4.

Figure 1 compares the evolution of chip and package sizes among three major packaging technologies: Package-on-Board (POB), Chip-on-Board (COB), and the proposed MiP4. Traditional POB designs exhibit the largest overall footprint, with individual LED packages exceeding 10,000 µm due to the need for separate encapsulation, reflectors, and substrates. COB architecture achieves moderate size reduction by directly bonding LED dies onto the system board; however, it still relies on relatively large sapphire-based chips and reflective cup structures to maintain brightness. In contrast, the MiP4 solution achieves substantial miniaturization, with chip dimensions of less than 500 µm and a package size of under 1000 µm. The inset image of the MiP4-based blue backlight demonstrates a dense and uniform emitter array, illustrating the high scalability and integration potential of this approach.

## 2. Experiment and Fabrication Process

This section outlines the fabrication process of the MiP4 chip, which integrates high-voltage μ-LED through chemical lift-off transfer and RDL routing on a glass substrate. The proposed process enables native 16 V operation at the chip level and SMT-ready packaging suitable for high-density Mini-LED backlight modules.

### 2.1. µ-LED Chip Transfer via Chemical Lift-Off Process

**Figure 2:**
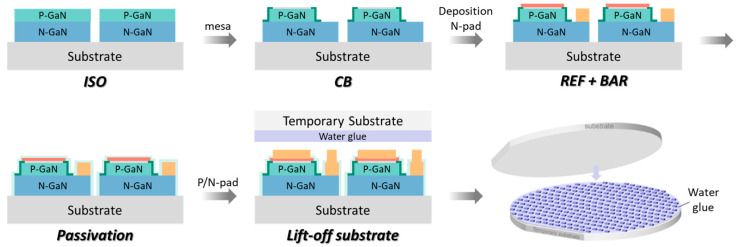
Process flow of µ-LED chip fabrication and chemical lift-off transfer with temporary bonding.

The fabrication of the MiP4 high-voltage µ-LED unit begins with the growth of GaN-based epitaxial layers on a sapphire substrate by metal–organic chemical vapor deposition (MOCVD). The epitaxial structure comprises an n-GaN layer, multiple quantum wells (MQWs), and a p-GaN layer, followed by deposition of transparent indium tin oxide (ITO) for current spreading and reflective metal layers to enhance light extraction. Figure 2 presents schematic illustrations of the fabrication flow for the µ-LED chip and chemical lift-off transfer process. The procedure begins with the fabrication of a front-end device on a GaN-on-sapphire wafer, which includes mesa etching, electrode formation, and sidewall passivation to define individual pixel structures. Device isolation (ISO) and mesa etching define the active emission regions, while a current blocking layer (CBL) is introduced to confine carrier injection and improve emission uniformity. N- and P-type electrode pads (N-pad and P-pad) are formed by metal deposition and patterning. A reflective metal mirror (REF) and barrier layer (BAR) are then added to improve both optical output and electrical contact stability. Sidewall passivation using Si_x_N_y_ dielectric is performed to suppress surface leakage and protect the exposed mesa edges. After completing front-side processing, a dual-metal PN electrode structure is defined to finalize the µ-LED device. To enable chip transfer, a sacrificial zinc oxide (ZnO) release layer is employed beneath the GaN epitaxial film. The ZnO layer is deposited prior to MOCVD growth, allowing the GaN-based epitaxy to be formed directly on top of the sacrificial layer. During chemical lift-off (CLO), an aqueous potassium hydroxide (KOH) solution is used to selectively dissolve the ZnO layer without damaging the GaN/InGaN epilayers, owing to the high etch selectivity between ZnO and GaN. This controlled undercutting process enables clean separation of the µ-LED film from the sapphire substrate while avoiding mechanical or laser-induced stress. The released μ-LEDs are temporarily bonded onto a carrier wafer coated with a water-soluble adhesive (water glue). This temporary substrate provides mechanical support for downstream processes, such as alignment, inspection, and handling. The wafer-level bonding approach also enables high-throughput parallel transfer, maintaining precise chip positioning across the entire wafer. Once transfer and testing are completed, the temporary carrier can be easily removed through water rinsing, leaving the µ-LED chips ready for permanent bonding to the target glass substrate, enabling subsequent transfer and integration steps.

### 2.2. RDL Routing for MiP4 Structure

Following the successful release and transfer of the chip, the µ-LED dies are permanently bonded to a glass-based substrate using either stamp or laser debonding. The glass carrier is pre-coated with an adhesion or catch layer that facilitates accurate chip placement and electrical interconnection. Compared with conventional ceramic or silicon carriers, the transparent glass substrate not only provides excellent thermal stability but also supports improved optical coupling and wide-angle emission for backlight applications.

**Figure 3:**
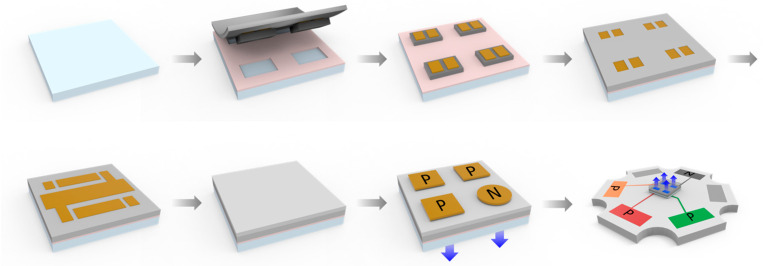
Schematic illustration of the fabrication sequence for MiP4 device formation on a glass substrate.

Figure 3 presents schematic illustrations of the fabrication sequence for MiP4 device formation on a glass substrate. The process begins with substrate preparation and cavity definition for μ-LED embedding, followed by precise chip transfer and alignment using a parallel stamp process. Subsequently, a polymer dielectric layer (polymer-1) is spin-coated and cured to form the RDL base. This layer electrically isolates the devices and provides the foundation for interconnect routing. A planarization step using polymer-2 is then applied to produce a smooth surface, minimizing topographical variations and improving metal adhesion uniformity. A patterned metal layer is deposited on top to connect the four µ-LED dies in series, forming the high-voltage MiP4 configuration. This 4-in-series connection allows native 16 V operation, thereby eliminating the need for external voltage step-up or step-down circuits in the backlight driver. Finally, top-side bump pads are fabricated to provide electrical input/output interfaces for subsequent SMT integration. The process concludes with encapsulation and inspection steps to ensure mechanical stability and electrical continuity. This chip-first, RDL-based integration approach enables compact, reliable, and optically transparent MiP4 modules that combine mechanical robustness, simplified interconnection, and compatibility with high-voltage driving architectures.

### 2.3. MiP4 Electrical Characterization

**Figure 4:**
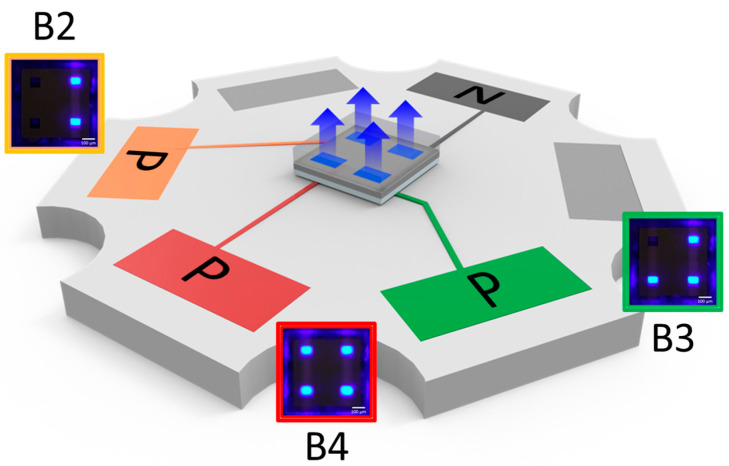
Schematic and optical images of dual- (B2), triple- (B3), and quad-chip (B4) MiP4 devices on an MCPCB substrate, showing uniform blue emission and successful series interconnection through the RDL structure.

To evaluate the electrical and optical performance of the MiP4 chip, the fabricated device was mounted on a metal-core starboard using standard die-bonding and wire-bonding techniques. Figure 4 presents schematic and optical images of the assembled structures, showing dual-, triple-, and quad-chip configurations (B2, B3, and B4) mounted on the metal-core substrate. This setup ensures mechanical stability, efficient heat dissipation, and convenient access for electrical probing during performance measurements. Under a forward bias of 16 V, the MiP4 device (B4) exhibited bright and uniform blue emission, confirming the successful four-in-series interconnection of µ-LED dies through the RDL routing structure. Further optical and electrical measurements were conducted on the B2, B3, and B4 configurations to systematically study voltage scalability, current uniformity, and emission efficiency as a function of the number of series-connected μ-LEDs. The B2 and B3 structures operated at nominal voltages of approximately 8 V and 12 V, respectively, while the B4 reached 16 V under forward bias.

The linear increase in operating voltage with respect to the number of connected chips confirms uniform current flow through each die and validates the electrical integrity of the RDL interconnects. All configurations exhibited consistent luminance across individual pixels, demonstrating reliable electrical routing, balanced current distribution, and excellent fabrication reproducibility.

## 3. Results and Discussion

### 3.1. Electrical and Optical Performance of Blue MiP4 Devices

**Figure 5:**
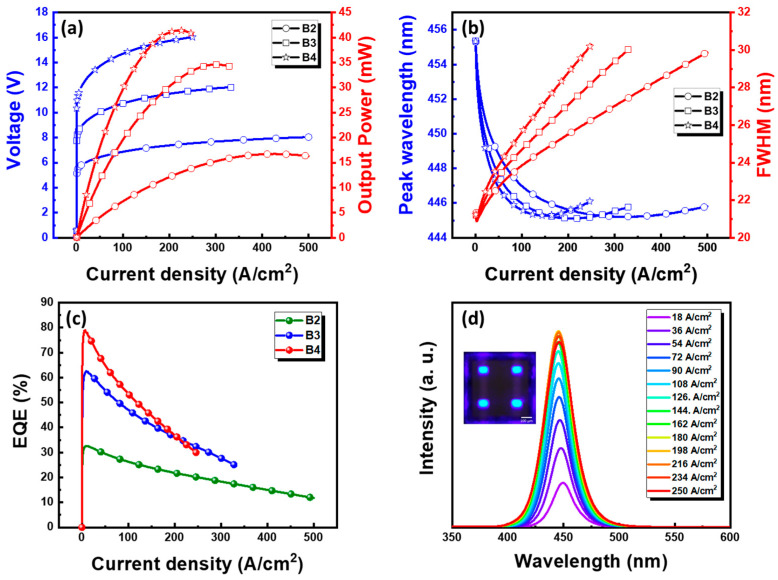
Electrical and optical characteristics of the blue MiP4 devices with dual- (B2), triple- (B3), and quad-chip (B4) configurations. (**a**) Voltage and output power as a function of current density. (**b**) Wavelength shift and FWHM variation with current density. (**c**) EQE versus current density. (**d**) EL spectra under different current densities.

Figure 5 presents the electrical and optical characteristics of the fabricated blue MiP4 devices with B2, B3, and B4 configurations, measured using a spectrometer (Maya 2000 Pro, Ocean Optics, Orlando, FL, USA). The voltage–current (I–V) curves exhibit clear rectifying behavior, with turn-on voltages of approximately 5.4 V, 8.1 V, and 10.6 V for B2, B3, and B4, respectively. At 200 A/cm^2^, the operating voltages scale linearly to 8 V, 12 V, and 16 V, confirming ideal voltage proportionality with the number of series-connected μ-LEDs. Correspondingly, the optical output powers reach 15.2, 27.6, and 42.3 mW, showing nearly linear optical scalability and negligible resistive loss through the RDL interconnects. Spectral measurements performed using an integrating sphere system (SLM-12, Isuzu Optics, Hsinchu, Taiwan) and a spectrometer (QE65000, Ocean Optics, Orlando, FL, USA) reveal a stable emission peak at 450 ± 2 nm with FWHM broadening from 22 to 29 nm due to slight junction heating. The external quantum efficiencies (EQE) of B2, B3, and B4 reach 78%, 63%, and 33% at 10 A/cm^2^ before exhibiting moderate efficiency droop at higher injection levels. These results confirm that the MiP4 architecture provides high-voltage operation, efficient optical scalability, and thermally stable emission, making it a strong candidate for compact and high-brightness Mini-LED backlight applications.

### 3.2. Comparison Between the Proposed MiP4 (B4) and a Commercial Blue LED

**Figure 6:**
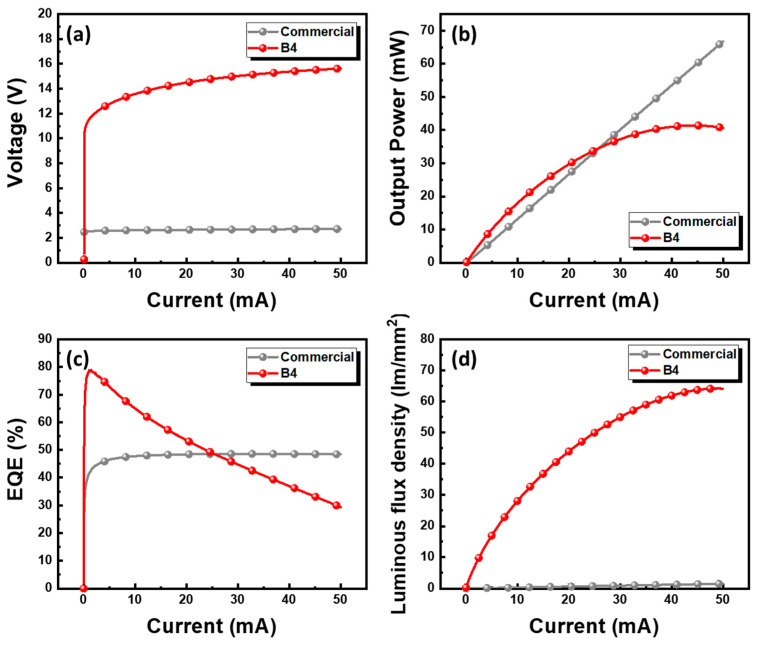
Electrical and optical performance comparison between the proposed MiP4 (B4) device and a commercial blue LED. (**a**) Current–voltage characteristics. (**b**) Optical output power as a function of current. (**c**) EQE versus current density. (**d**) Luminous flux density comparison between the two devices.

**Figure 7:**
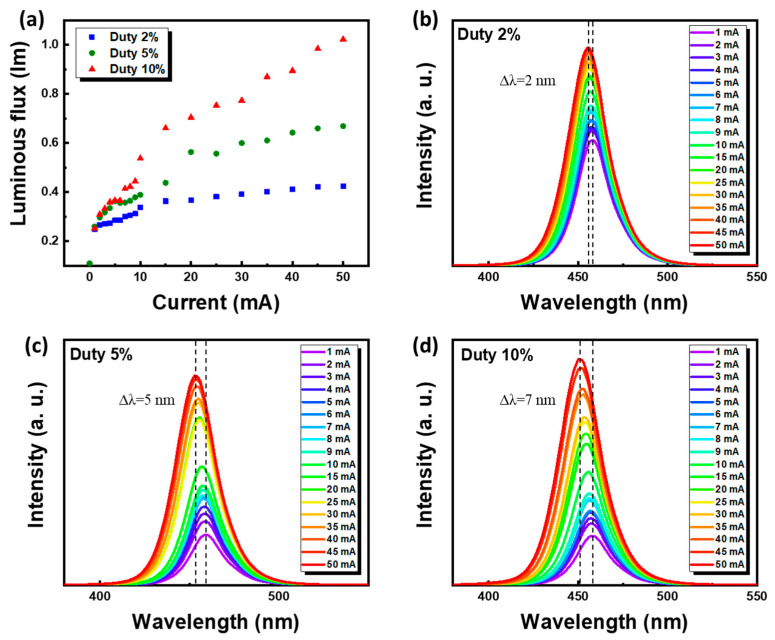
Pulse-driven optical characteristics of the MiP4 (B4) device under different duty cycles. (**a**) Optical output power versus current under 2%, 5%, and 10% duty cycles. EL spectra of the MiP4 under (**b**) 2%, (**c**) 5%, and (**d**) 10% duty cycles.

To benchmark the performance of the proposed MiP4 device, its electrical and optical behaviors were compared with a commercial blue LED, as shown in Figure 6. In Figure 6a, both devices exhibit typical rectifying I–V characteristics, while the MiP4 shows a significantly higher forward voltage of approximately 15.2 V at 20 mA, owing to its four-in-series configuration. The higher operating voltage enables the device to be driven efficiently by high-voltage backlight circuits, eliminating the need for DC-DC conversion and resulting in lower overall power consumption and a simplified driver design. As shown in Figure 6b, the optical output power increases monotonically with current for both devices. Around 25 mA, the MiP4 and the commercial LED exhibit comparable optical power (~35 mW). However, the MiP4’s active emitting area (~20,000 µm^2^) is nearly two orders of magnitude smaller than that of the commercial device (~1,350,000 µm^2^). This demonstrates the MiP4’s outstanding luminance efficiency per unit area, making it particularly advantageous for high-resolution or fine-pitch backlight applications where chip size is a critical constraint. In Figure 6c, the EQE versus current plot shows that the MiP4 maintains high EQE at low injection levels and exhibits only moderate efficiency droop at higher currents, indicating effective carrier confinement and reduced resistive loss through the RDL interconnects. However, a monotonic decrease in EQE is observed with increasing current, eventually causing the EQE of the MiP4 to fall below that of the commercial counterpart at higher injection levels. This phenomenon is attributed to the significantly smaller emission area of the MiP4, which results in a much higher current density under the same injection current. This elevated current density enhances efficiency droop mechanisms, such as Auger recombination and carrier overflow, leading to a more rapid decline in EQE. Additionally, the four-in-series configuration introduces a larger overall series resistance, and the compact device geometry contributes to localized thermal accumulation during DC operation. The increased Joule heating reduces the radiative recombination efficiency, contributing to the ultimate crossover in EQE performance. Nevertheless, despite the lower EQE at high currents, the MiP4 still delivers a substantially higher luminous flux density, a crucial metric for high-power-density applications, due to its significantly smaller chip footprint. As further verified by the pulse-driven results in Figure 7, the efficiency degradation is greatly mitigated when thermal effects are suppressed, confirming that the EQE trend is dominated by current-density- and thermal-related mechanisms rather than intrinsic material limitations.

Most importantly, Figure 6d compares the luminous flux density (optical flux normalized by emission area) between the two devices. Across the entire current range, the MiP4 exhibits a substantially higher luminous flux density, demonstrating its superior optical intensity and power density under the same driving current. In particular, at 50 mA, the B4 device achieves a luminous flux density of approximately 64 lm/mm^2^, which is several times higher than that of the commercial LED. This remarkable enhancement confirms the effectiveness of the glass-based RDL architecture in realizing compact, high-brightness, and high-efficiency light-emitting units suitable for next-generation Mini-LED backlight systems.

### 3.3. Pulse-Driven Performance Under Different Duty Cycles

To further evaluate the transient response and thermal stability of the MiP4 (B4) device, pulse-mode measurements were performed under duty cycles of 2%, 5%, and 10% with varying injection currents ranging from 1 to 50 mA. In these measurements, the MiP4 was driven by an ILX LDP-3840B (ILX Lightwave, Bozeman, MT, USA) pulse current source with a fixed pulse width of 0.5 µs. The corresponding pulse periods (PRI) and repetition frequencies were set to 0.025 ms (40 kHz), 0.01 ms (100 kHz), and 0.005 ms (200 kHz) for 2%, 5%, and 10% duty cycles, respectively, ensuring that the thermal loading conditions were well-controlled during the pulse operation.

As shown in Figure 7a, the optical output power increases proportionally with both current and duty cycle. Devices driven under higher duty ratios exhibit stronger emission intensity due to extended current-on time, whereas the lower duty-cycle operation effectively suppresses junction heating, maintaining consistent optical efficiency. The combination of high peak brightness and low thermal load confirms the suitability of MiP4 for high-speed and high-frequency display driving schemes. Figure 7b–d present the EL spectra of the MiP4 under different duty conditions. The emission peak remains centered at approximately 450 nm across all pulse modes, showing negligible wavelength shift (<1 nm) and FWHM variation (<2 nm) even at 50 mA. These results indicate excellent thermal and spectral stability, which can be attributed to efficient heat dissipation through the transparent glass carrier and uniform current spreading via the RDL interconnects. Overall, the MiP4 demonstrates robust pulse-driven performance, combining high optical output, stable spectral characteristics, and superior energy efficiency—critical attributes for next-generation Mini-LED backlight and visible-light communication applications.

## 4. Conclusions

In this work, a unique 4-in-1 MiP4 architecture was successfully developed, integrating four sub-85 µm blue micro-LED dies in series on a glass substrate via RDL interconnection. The resulting device achieves native 16 V operation, compact size (0.6 mm × 0.6 mm), and excellent current uniformity without external voltage conversion. The measured optical power scales linearly with chip count, reaching 42.3 mW for B4 at 200 A cm^−2^, with a stable emission peak at 450 ± 2 nm. Compared with a commercial blue LED, the MiP4 demonstrates 1.8 times higher optical output and a significantly greater luminous flux density of 64 lm mm^−2^ at 50 mA, despite using only ~1/70 of the chip area. Under pulse-driven operation (2%, 5%, and 10% duty), the MiP4 maintained excellent spectral stability (<1 nm shift) and consistent optical efficiency, confirming robust transient and thermal performance. These results validate the effectiveness of the glass-based RDL architecture in achieving high-brightness, high-efficiency micro-LEDs within a miniaturized footprint. This first demonstration of a 4-in-1, high-voltage, glass-integrated micro-LED package establishes a new paradigm for next-generation mini-LED backlights, AR/VR displays, and optical interconnect applications.

## Data Availability

The data presented in this study are available from the corresponding author upon reasonable request.
